# Acute Cardiovascular Complications of COVID-19: A Systematic Review

**DOI:** 10.7759/cureus.38576

**Published:** 2023-05-05

**Authors:** Oluwaremilekun Tolu-Akinnawo, Frank Adusei Poku, Thomas Elimihele, Matthew League, Caleb F Adkins, Henry Okafor

**Affiliations:** 1 Internal Medicine, Meharry Medical College, Nashville, USA; 2 Medicine, Lincoln Memorial University-DeBusk College of Osteopathic Medicine, Knoxville, USA; 3 Cardiology, Vanderbilt University Medical Center, Nashville, USA

**Keywords:** pericardial tamponade, takotsubo cardiomyopathy, thrombotic complications of covid-19, covid-induced myocarditis, viral pericarditis, acute myocardial infarct, covid-19-induced arrhythmia, covid and heart, covid 19

## Abstract

Since the pandemic in 2019, coronavirus 2019 (COVID-19) has continued to be linked with a variety of organ systems and complications. While it is generally considered a respiratory disease, its link with the heart is widely discussed in the literature. This article focuses on the acute cardiovascular complications of COVID-19 and the possible predictors of these complications. Our study included 97 articles (58 case reports, eight case series, 23 retrospective cohort studies, five prospective cohort studies, and three cross-sectional studies). Several mechanisms have been proposed to explain COVID-19-induced cardiovascular complications, with cytokine-induced inflammation and direct cardiac damage noted as the significant focus. Patients with underlying cardiovascular complications such as hypertension and diabetes were noted to be at increased risk of acute cardiovascular complications, as well as an increased risk of severe disease and death. Also, acute myocardial infarction and arrhythmias were two of the most common acute cardiovascular complications noted in our review. Other acute cardiovascular complications are myocarditis, takotsubo syndrome, acute thromboembolic events, and pericardial complications. This article provides an updated review of acute cardiovascular complications of COVID-19, its pathogenesis, and risk stratification and emphasizes the need for high suspicion in patients with underlying cardiovascular risk factors.

## Introduction and background

The coronavirus disease 2019 (COVID-19) remains one of the most fatal pandemics the world has experienced recently. The emergence of COVID-19 was first described in a group of patients presenting in Wuhan, China, with severe pneumonia-like symptoms (first wave). In this subset of patients, a novel virus called the severe acute respiratory syndrome coronavirus-2 (SARS-CoV-2) was isolated from the lower respiratory tract samples [1]. Since then, at least five outbreak waves have been described [2]. The first wave presented majorly with respiratory symptoms, while gastrointestinal symptoms were added during the second wave, and peripheral neurological manifestations replaced the gastrointestinal symptoms in the third wave. Central nervous system manifestations were added to the fourth and fifth waves [2]. However, key to note that as the pandemic wore on, morbidity and mortality have continued to increase, with cardiovascular complications a peak of the group. So far, in the United States, there have been 103,499,382 confirmed cases and 1,117,856 confirmed deaths from COVID-19-related complications [3]. Although most presentations have been related to varying severity of upper and lower respiratory system involvement, evidence of extrapulmonary manifestations remains equally common [4, 5]. Several cardiac complications, such as acute cardiac injury, heart failure, arrhythmias, cardiogenic shock, and right ventricular dysfunction, have been documented in current literature, with significant impacts on outcomes reported [6]. Direct myocardial cell injury (via the ACE2 receptors), overwhelming systemic inflammation, hypoxic state, catecholamines storm, cytokines release, and electrolyte derangements have been suggested as the possible link between COVID-19 and cardiovascular complications [7]. Literature has also noted an increased risk of cardiovascular events in patients with pre-existing cardiovascular diseases such as hypertension and diabetes. This systematic review aims to describe the acute cardiovascular complications of COVID-19, the outcome reported in the literature so far, and the associated risk factors, such as in patients with existing co-morbidities. This review article also aims to provide awareness of cardiovascular events in patients with COVID-19, promoting the suspicion of cardiac events for early identification and intervention.

## Review

Methods

Search Strategy

**Figure 1:**
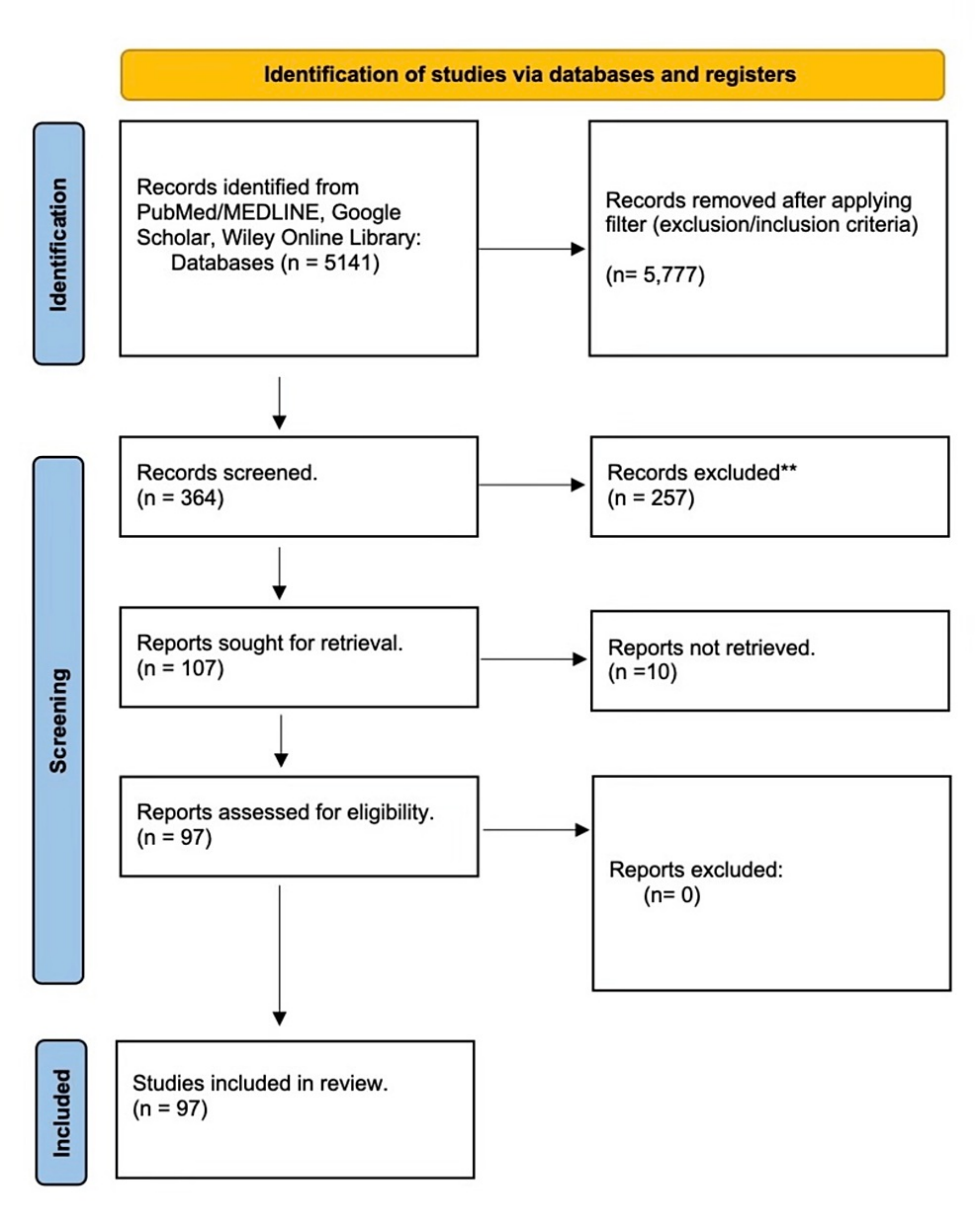
PRISMA Flowchart of Selected Articles. PRISMA: Preferred Reporting Items for Systematic Reviews and Meta-Analyses; MEDLINE: Medical Literature Analysis and Retrieval System Online.

We searched databases (PubMed/MEDLINE, Google Scholar, Wiley Online Library) using free-text terms in the title and appropriate Medical Subject Headings (MeSH) terms- COVID-19 and Acute cardiovascular complications. We collected all records into one library, with 364 results found. We exported the final list into a Word document file to remove duplicates. All search results, including titles and abstracts of retrieved articles, were then screened to assess for eligibility which was independently reviewed and agreed upon by team consensus. Finally, we obtained a full-length manuscript for all intended studies for review, which was 97 articles (58 case reports, 8 case series, 23 retrospective cohort studies, 5 prospective cohort studies, and 3 cross-sectional studies). The Preferred Reporting Items for Systematic Reviews and Meta-Analyses (PRISMA) flowchart shown in Figure 1 below further highlights the process used in our articles selection.

Inclusion and Exclusion Criteria

We assessed primary studies of any design type, including randomized clinical trials, case-control, prospective and retrospective cohort studies, case series, and reports published between January 2020 and September 2022, evaluating acute cardiovascular complications in COVID-19 patients and associated clinical risk factors. No restrictions were made with regard to the age, sex, race, ethnicity, nationality, or vaccination status of the individuals.

We included studies that have indexed COVID-19 disease as confirmed by rt-PCR. Acute cardiovascular complications are cerebrovascular disorders, dysrhythmias, ischemic and non-ischemic heart disease, inflammatory heart conditions (pericarditis and myocarditis), cardiomyopathies, myocardial infarction (MI), heart failure, and thromboembolic disease.

We excluded systematic reviews and meta-analyses; international expert recommendations; studies with non-human subjects or in vitro studies; studies with data not reliably extracted, duplicate, or overlapping data; abstract-only papers as preceding papers, conference, editorial, and author response theses and books; and articles without available full text.

Objectives

The primary objective of this review is to describe and analyze the acute cardiovascular complications reported in patients with COVID-19 and to identify the potential underlying risk factors (i.e., diabetes mellitus, hypertension, hyperlipidemia, tobacco use, coronary heart disease, previous cerebrovascular accident, COPD, malignancy, chronic kidney disease, chronic liver disease) associated with developing these cardiovascular complications. Secondary objectives include analysis of the effects of acute cardiovascular complications on mortality and its impact on clinical decision-making in managing COVID-19 patients.

Results

Electronic literature searches identified 364 articles in total. We excluded systematic reviews and meta-analyses; international expert recommendations; studies with non-human subjects or in vitro studies; studies with data not reliably extracted, duplicate, or overlapping data; abstract-only papers as preceding papers, conference, editorial, and author response theses and books; and articles without available full text. We selected 97 articles for full-text review pertaining to our study.

Below are tables (Tables 1-7) showing articles reporting each of our primary outcome data: Acute cardiovascular complications of COVID.

**Table 1:** Cases of COVID-induced Type 1 Myocardial Infarction, Coronary Artery Thrombosis, and Coronary Artery Dissection. HTN: Hypertension, DM: Diabetes Mellitus, MI: Myocardial Infarction, s/p: Status Post, N/A: Not available - data not provided in the study.

Cardiovascular manifestation	Study authors	Type of Study	Age at diagnosis	Underlying comorbidity	Outcome
Type 1 MI or coronary thrombosis	Sheikh et al. [8]	Case report	56 years	CAD, HLD, HTN	One patient recovered/discharged post-intervention
Type 1 MI or coronary thrombosis	Juthani et al. [9]	Case Report	29 years	None	One patient recovered/discharged post-intervention
Type 1 Myocardial Infarction/Coronary Thrombosis	Wolsky and Bateman [10]	Case Report	26 years	None	One patient recovered/ discharged post-intervention
Type 1 Myocardial Infarction/Coronary Thrombosis	Lalani et al. [11]	Retrospective cohort	N/A	Hypertension	26 out of 73 (35%) were COVID-19-positive
Type 1 Myocardial Infarction/Coronary Thrombosis	Matsuda et al. [12]	Case Report	43 years	Hypertension, Hyperlipidemia, Smoking	One patient recovered/discharged post-intervention
Type 1 Myocardial Infarction/ Coronary Thrombosis	Montaseri et al. [13]	Case series	50 years	None	Of three patients, 1 out of three died (33.3%), two recovered post-intervention
			70 years	Diabetes	
			54 years	None	
Type 1 Myocardial Infarction/Coronary Thrombosis	Mandal et al. [14]	Case Report	82 years	Diabetes	One patient recovered/discharged post-intervention
Type 1 Myocardial Infarction/ Coronary Thrombosis	Cuevas et al. [15]	Case Report	39 years	None	One patient recovered/discharged post-intervention
Type 1 Myocardial Infarction/Coronary Thrombosis	Abe et al. [16]	Retrospective	N/A	Diabetes versus non-diabetic	9.9% vs 1.4% of patients (P=0.03) respectively
Type 1 Myocardial Infarction/ Coronary Thrombosis	Valchanov et al. [17]	Case Report	43 years	Smoker	One patient died
Type 1 Myocardial Infarction/Coronary Thrombosis	Tschöpe et al. [18]	Case Report	74 years	Hypertension	One patient recovered post-intervention
Type 1 Myocardial Infarction/Coronary Thrombosis	Pelle et al. [19]	Case Report	88 years	Hypertension	One patient recovered post-intervention
Type 1 Myocardial Infarction/Coronary Thrombosis	Ali et al. [20]	Case Report	59 years	Diabetes	One patient
Type 1 Myocardial Infarction/Coronary Thrombosis	Monica et al. [21]	Prospective	75 years and above	N/A	Five patients (1.6%). Some survived, while some died.
Type 1 Myocardial Infarction/Coronary Thrombosis	Jalali et al. [22]	Retrospective	65 years	N/A	12 patients (6.1%), varying outcome
Type 1 Myocardial Infarction/Coronary Thrombosis	Kaeley et al. [23]	Case series	45 years	Diabetes	One out of four (25%) patients who had MI died.
Type 1 Myocardial Infarction/Coronary Thrombosis	Boudihi et al. [24]	Case report	54 years	Diabetes	One patient recovered/discharged post-intervention.
Type 1 Myocardial Infarction/Coronary Thrombosis	Sharma et al. [25]	Case Report	54 years	Hypertension, Hyperlipidemia, Obesity	One patient recovered/discharged post-intervention.
Type 1 Myocardial Infarction/Coronary Thrombosis	Siddamreddy et al. [26]	Case Report	61 years	CAD, Diabetes Hypertension	One patient recovered/discharged post-intervention.
Type 1 Myocardial Infarction/Coronary Thrombosis	Shuroog et al. [27]	Case Report	72 years	Complete heart block s/p pacemaker	One patient recovered.
Type 1 Myocardial Infarction/Coronary Thrombosis	Mohamed et al. [28]	Case Report	55 years	None	One patient recovered/discharged post-intervention
Type 1 Myocardial Infarction/Coronary Thrombosis	Hashemi et al. [29]	Case Report	70 years	Diabetes, Hypertension	One patient died.
Type 1 Myocardial Infarction/Coronary Thrombosis	Meizinger and Klugherz [30]	Case Report	86 years	Hypertension, Dementia	One patient died.
Type 1 Myocardial Infarction/Coronary Thrombosis	Genovese et al. [31]	Case Report	60 years	Lumbar radiculopathy	One patient died.
Type 1 Myocardial Infarction/Coronary Thrombosis	Demertzis et al. [32]	Case Report	67 years	Non-ischemic cardiomyopathy	One patient died.
Type 1 Myocardial Infarction/Coronary Thrombosis	Linschoten et al. [33]	Retrospective	67 years	Arrhythmias (15.1%), coronary artery disease (15.4%)	11.5% of patients, varying mortality.
Coronary dissection	Kireev et al. [34]	Case Report	35 years	None	One patient recovered.
Coronary dissection	Yapan et al. [35]	Case Report	50 years	None	One patient recovered.

**Table 2:** COVID-19-induced Heart failure, Cardiogenic Shock Cases. N/A: Not available - Data not provided.

Cardiovascular Manifestation	Study author	Type of Study	Age at diagnosis	Underlying co-morbidity	Outcome
Heart Failure, Cardiogenic Shock, Right Ventricular Dysfunction	Xiong et al. [36]	Retrospective	Median age 64 years	Hypertension (38.8%), diabetes (16.4%), coronary heart disease (14.7%)	18 (32.7%) patients, the outcome not discussed.
Heart Failure, Cardiogenic Shock, Right Ventricular Dysfunction	Jacobs et al. [37]	Case Report	48 years	None	Cardiogenic shock >> Intensive Care Unit, the outcome not specified.
Heart Failure, Cardiogenic Shock, Right Ventricular Dysfunction	Valiton et al. [38]	Case Report	52 years	Non-disabling Raynaud syndrome	One patient, recovered and discharged.
Heart Failure, Cardiogenic Shock, Right Ventricular Dysfunction	Abe et al. [16]	Retrospective	N/A	Diabetes versus non-diabetic	25.3% of patients with diabetes versus 5.6% of non-diabetes developed acute heart failure. Specific outcome not discussed.
Heart Failure, Cardiogenic Shock, Right Ventricular Dysfunction	Linschoten et al. [33]	Retrospective	Median Age 67 years	Arrhythmias (15.1%), coronary artery disease (15.4%)	15% developed acute heart failure and recovered.
Heart Failure, Cardiogenic Shock, Right Ventricular Dysfunction	Kaeley et al. [23]	Case Series	20 years	Pregnant	One patient, acute heart failure recovered and was discharged.
Heart Failure, Cardiogenic Shock, Right Ventricular Dysfunction	Chen et al. [39]	Case Report	73 years	Diabetes, hypertension, hyperlipidemia	Cor-pulmonale, ICU>> died.

**Table 3:** Cases of COVID-19-induced Myocarditis. N/A: Not available - data not provided.

Cardiovascular Manifestation	Study author	Type of Study	Age at diagnosis	Underlying co-morbidity	Outcome
Myocarditis	Fischer et al. [40]	Case Report	15 years	None	One patient, ICU>> Improved
Myocarditis	Ismayl et al. [41]	Case Report	53 years	None	One patient, multiorgan failure>>improved
Myocarditis	Abe et al. [16]	Retrospective	N/A	Diabetes versus non-diabetic	36.6% of diabetes developed acute myocarditis versus 15.5% of non-diabetic.
Myocarditis	Shen et al. [42]	Case Report	43 years	None	One patient recovered and was discharged.
Myocarditis	Jalali et al. [22]	Retrospective	Median age 65 years	N/A	14 (7.7%) of patients had myocarditis
Myocarditis	Zeng et al. [43]	Case Report	63 years	None	One patient died.
Myocarditis	Ghafoor et al. [44]	Case Report	54 years	Hypertension, heart failure	One patient died.
Myocarditis	Kallel et al. [45]	Case Series	26 years	None	One patient recovered.
			56 years	Diabetes	One patient recovered.
Myocarditis	Finn et al. [46]	Retrospective	N/A	Absent in 29.3% of cases.	46.3% with acute myocarditis, a mortality rate of 61%
Myocarditis	Pascariello et al. [47]	Case Report	19 years	None	One patient recovered.

**Table 4:** Cases of COVID-induced Pericarditis, Pericardial Effusion, and Cardiac Tamponade N/A: Not available - data not provided in the study.

Cardiovascular Manifestation	Study author	Type of Study	Age at diagnosis	Underlying co-morbidity	Outcome
Pericarditis, Pericardial Effusion and Cardiac Tamponade	Derveni et al. [48]	Case Report	89 years	Chronic Obstructive Pulmonary Disease (COPD)	One patient, cardiac tamponade>>died.
Pericarditis, Pericardial Effusion and Cardiac Tamponade	Kumar et al. [49]	Case Report	67 years	None	One patient, cardiac tamponade>>recovered.
Pericarditis, Pericardial Effusion and Cardiac Tamponade	Mir et al. [50]	Case Series	56 years	Diabetes	One patient recovered.
			56 years	None	One patient recovered.
Pericarditis, Pericardial Effusion and Cardiac Tamponade	Kim et al. [51]	Retrospective	N/A	N/A	5 patients (8.7% of patients) >> pericardial effusion
Pericarditis, Pericardial Effusion and Cardiac Tamponade	Essa and Ahmed [52]	Case Report	54 years	None	One patient, cardiac tamponade>>recovered.
Pericarditis, Pericardial Effusion and Cardiac Tamponade	Abdelsayed et al. [53]	Case Report	57 years	Hypertension	One patient, hemorrhagic pericardial effusion>> recovered.
Pericarditis, Pericardial Effusion and Cardiac Tamponade	Heidari et al. [54]	Case Report	28 years	None	One patient with massive hemorrhagic cardiac tamponade>>recovered.
Pericarditis, Pericardial Effusion and Cardiac Tamponade	Rivera-Morales et al. [55]	Case Report	73 years	Hypertension	One patient, pericardial effusion>>recovered.
				Dyslipidemia	
				Diabetes	
Pericarditis, Pericardial Effusion and Cardiac Tamponade	Brockman et al. [56]	Case Report	71 years	Hepatitis C	One patient, pericarditis>>recovered.
				Diabetes	
				Asthma	
Pericarditis, Pericardial Effusion and Cardiac Tamponade	Sayad et al. [57]	Case Report	13 years	Congenital sideroblastic anemia	1 patient, pericardial effusion>>respiratory failure/intubation>>died.
Pericarditis, Pericardial Effusion and Cardiac Tamponade	Okor et al. [58]	Case Report	72 years	Hypertension	One patient, pericarditis>> died.
				COPD on chronic 2L oxygen	
Pericarditis, Pericardial Effusion and Cardiac Tamponade	Afriyie et al. [59]	Case Report	27 years	None	One patient, fulminant myopericarditis >> died.

**Table 5:** Cases of COVID-19-induced Thromboembolism. CAD: Coronary Artery Disease, PAD: Peripheral Artery Disease, DM: Diabetes Mellitus, DVT: Deep Venous Thrombosis, PE: Pulmonary Embolism, N/A: Not available - data not provided in the study.

Cardiovascular Manifestation	Study author	Type of Study	Age at diagnosis	Underlying co-morbidity	Outcome
Thromboembolism	Kaki et al. [60]	Case Report	57 years	Asthma	1 patient, large intracardiac thrombosis>>thrombectomy >>recovered.
Thromboembolism	Castillo-Garcia et al. [61]	Case Report	70 years	Diabetes	1 patient, pulmonary embolism>>surgical thrombectomy>>amputation.
Thromboembolism	Bois et al. [62]	Retrospective	N/A	N/A	12 of 16 (75%) cases of post-mortem patients had COVID.
Thromboembolism	Monica et al. [21]	Prospective	75 years and above	Renal Failure	17 (5.6%) patients had a pulmonary embolism (PE).
				Congestive Heart Failure	
Thromboembolism	Jalali et al. [22]	Retrospective	65 years	N/A	4 (2%) had deep vein thrombosis (DVT).
Thromboembolism	Kaeley et al. [23]	Case Series	62 years	N/A	1 patient, deep venous thrombosis & bilateral pulmonary embolism>>died.
Thromboembolism	Chaughtai et al. [63]	Case Report	49 years	None	One patient had a large vessel stroke>> and recovered.
Thromboembolism	Hughes et al. [64]	Case Report	59 years	Hypertension	One patient, stroke>>recovered.
Thromboembolism	Lodigiani et al. [65]	Prospective	66 years	N/A	28 (7.7%) patients with thromboembolism.
Thromboembolism	Katsoularis et al. [66]	Retrospective	N/A	N/A	The odds ratio of stroke in COVID-19 patients is 3.63 (1.69-7.80)
Thromboembolism	Brem et al. [67]	Case Report	65 years	Hypertension, Diabetes	1 patient, acute limb ischemia>>amputation>>died.
Thromboembolism	Tang et al. [68]	Retrospective	54 years	41% with chronic medical disease	Disseminated intravascular coagulation was present in 7.4% of non-survivors vs. 0.6% of survivors.
Thromboembolism	Yu et al. [69]	Case Report	55 years	Hypertension, Diabetes	1 patient, pulmonary embolism>>Myocardial infarction>> recovered.
Thromboembolism	Genovese et al. [31]	Case Report	60 years	Lumbar radiculopathy	1 patient, right coronary thrombosis>>myocardial infarction>>died.
Thromboembolism	Wichmann et al. [70]	Prospective cohort	Median age ~ 73 years	Obesity, CAD, DM, Asthma, COPD, PAD	7 out of 12 patients (58%) had a DVT. 4 out of 12 patients died directly of PE.

**Table 6:** Cases of COVID-19-induced Takotsubo Cardiomyopathy.

Cardiovascular Manifestation	Study author	Type of Study	Age at diagnosis	Underlying co-morbidity	Outcome
Takotsubo cardiomyopathy	Demertzis et al. [32]	Case Series	67 years	Non-ischemic cardiomyopathy	One patient, Takotsubo syndrome, recovered.
			76 years	Hypertension	One patient with Takotsubo syndrome died.
Takotsubo cardiomyopathy	Meyer et al. [71]	Case Report	83 years	Hypertension	One patient recovered.
Takotsubo cardiomyopathy	Arroyo-Rodriguez et al. [72]	Case Series	74 years	Hypertension	One patient died.
			76 years	Diabetes, smoker	One patient recovered.
			66 years	Hypertension, dyslipidemia	1 patient, Takotsubo>>cardiogenic shock, died.
			65 years	Hypertension, diabetes, dyslipidemia	One patient died.
			75 years	Hypertension, previous ischemic stroke, chronic kidney disease	1 patient, Takotsubo>>cardiogenic shock, died.
Takotsubo cardiomyopathy	Okor et al. [73]	Case Report	72 years	Paroxysmal atrial fibrillation	One patient recovered.
Takotsubo cardiomyopathy	Namburu et al. [74]	Case Report	69 years	Hypertension	One patient recovered.
Takotsubo cardiomyopathy	Belli et al. [75]	Case Report	53 years	Stage III Chronic Kidney Disease	One patient recovered.
Takotsubo cardiomyopathy	Roca et al. [76]	Case Report	87 years	Breast cancer	One patient recovered.

**Table 7:** Cases of COVID-19-induced Arrhythmias. HTN: Hypertension, CAD: Coronary Artery Disease, DM: Diabetes Mellitus, CKD: Chronic Kidney Disease, HFpEF: Heart Failure with Preserved Ejection Fraction, N/A: Not available - Data not provided in the article.

Cardiovascular Manifestation	Study author	Type of Study	Age at diagnosis	Underlying co-morbidity	Outcome
Arrhythmias	Babapoor-Farrokhran et al. [77]	Case Report	69 years	Hypertension, Hyperlipidemia, Diabetes, Ischemic stroke	1 patient, 2:1 AV Block>>Resolved.
			83 years	Hypertension, Hyperlipidemia	1 patient, sinus arrest>>non-sustained ventricular tachycardia>>sustained supraventricular tachycardia>>resolved.
Arrhythmias	Radwan and Schwartz [78]	Case Report	37 years	None	One patient, new-onset atrial fibrillation.
Arrhythmias	Wiemken et al. [79]	Retrospective	N/A	N/A	Dysrhythmia is the most common cardiovascular outcome.
Arrhythmias	Lalani et al. [11]	Retrospective	N/A	Hypertension, Diabetes, Chronic Kidney Disease (CKD).	45.6% of 730 patients with prolonged QTc interval, sinus tachycardia in 24.2%.
Arrhythmias	Amaratunga et al. [80]	Case Series	55 years	Hypothyroidism	The four patients developed new-onset sinus bradycardia.
			60 years	None	
			78 years	Hypothyroidism, CAD	
			73 years	CAD, HTN, HLD	
Arrhythmias	Khawaja et al. [81]	Retrospective	67.4 +/- 16.1 years	Hypertension (53.4%), Diabetes (40.2%), Hyperlipidemia (31.7%), History of CAD (13.9%), Stroke (11.8%), Heart Failure (6.6%)	19.1% with new onset atrial fibrillation.
Arrhythmias	Doodnauth et al. [82]	Case Report	84 years	Hypertension, Diabetes, HFpEF, CAD, CKD	1 patient>>sinus bradycardia>>dual chamber permanent pacemaker.
Arrhythmias	Abe et al. [16]	Retrospective	N/A	Diabetic versus Non-diabetic	12.7% had new-onset atrial fibrillation in diabetics vs. 1.4% in non-diabetics.
Arrhythmias	Altuwaijri and Alotaibi [83]	Case Report	64 years	Hypertension and diabetes	One patient had ventricular fibrillation.
Arrhythmias	Linschoten et al. [33]	Retrospective	67 years	CAD	11.5% with new-onset arrhythmias/conduction disorders.
Arrhythmias	Monica et al. [21]	Prospective	> 75 years	N/A	New-onset atrial fibrillation in 20 (6.5%) of patients.
Arrhythmias	Jalali et al. [22]	Retrospective	65 years	N/A	Arrhythmias is the most common complication, 2 in 9 (14.8%) patients.
Arrhythmias	Kaeley et al. [23]	Case Series	65 years	None	25% of patients had new-onset right bundle branch block (RBBB) with fascicular block>>improved.
Arrhythmias	Hakami et al. [84]	Case Series	34 years	None	One patient with bradyarrhythmia >> resolved.
			34 years	Tobacco dependence	1 patient with prolonged PR/QT/U interval-bradyarrhythmia>>resolved.
Arrhythmias	Bhatia et al. [85]	Retrospective	N/A	N/A	3.6% developed new-onset atrial fibrillation or flutter, and 0.5% developed high-grade AV block.
Arrhythmias	Al-Zakhari et al. [86]	Retrospective	N/A	N/A	New-onset arrhythmia, severity not specified.
Arrhythmias	Hopkins and Webster [87]	Case Report	Nine days old	None	One patient, supraventricular tachycardia>>resolved.
Arrhythmias	Olagunju et al. [88]	Case Report	55 years	Morbid obesity	One patient had multiple sinus pauses.

Discussion

COVID-19 and Patients With Underlying Cardiovascular Disease or Cardiovascular Disease Risk Factors

Literature has shown that patients with pre-existing cardiovascular disease or cardiovascular disease risk factors are at risk for more severe COVID-19 complications. In a study by Shao et al., hypertension was noted in approximately 55.6% of COVID-19 cases [89]. In a study by Cao et al., hypertension was found in 55% of ICU patients with COVID-19 [90]. In a study by Chen et al., 48% of deceased patients from COVID-19 had underlying hypertension, while only 24% of the recovered patients had hypertension [91]. Also, in a study by Zhou et al., hypertension was found in 23% versus 48% (P=0.0008) of survivors and non-survivors, respectively [92]. Also, hypertension (38%, P = 0.01) was noted to be the most common comorbidity found in patients who developed acute cardiac injury during hospital stay [93].

In a study by Chen et al., underlying cardiovascular disease was more common in patients who died versus those that survived (14% versus 4%) [91]. Zhang et al. also noted that underlying cardiovascular disease was present in severe COVID-19 presentations (23.6%) versus mild COVID-19 manifestations (5.4%, P < 0.001) [94]. In a study by Guo et al., patients with underlying cardiovascular disease (such as coronary heart disease, cardiomyopathy, underlying arrhythmia, and hypertension) and developed cardiac injury had higher mortality rates at up to 69.44% versus 13.3% in patients without underlying cardiovascular disease [95].

The above findings emphasize the link between COVID-19 and underlying cardiovascular disease in predicting the severity of COVID-19 outcomes. Patients with pre-existing cardiovascular disease or cardiovascular disease risk factors have an increased rate of severe presentation, critical care unit admission, and higher mortality rates.

Pathophysiology

**Figure 2:**
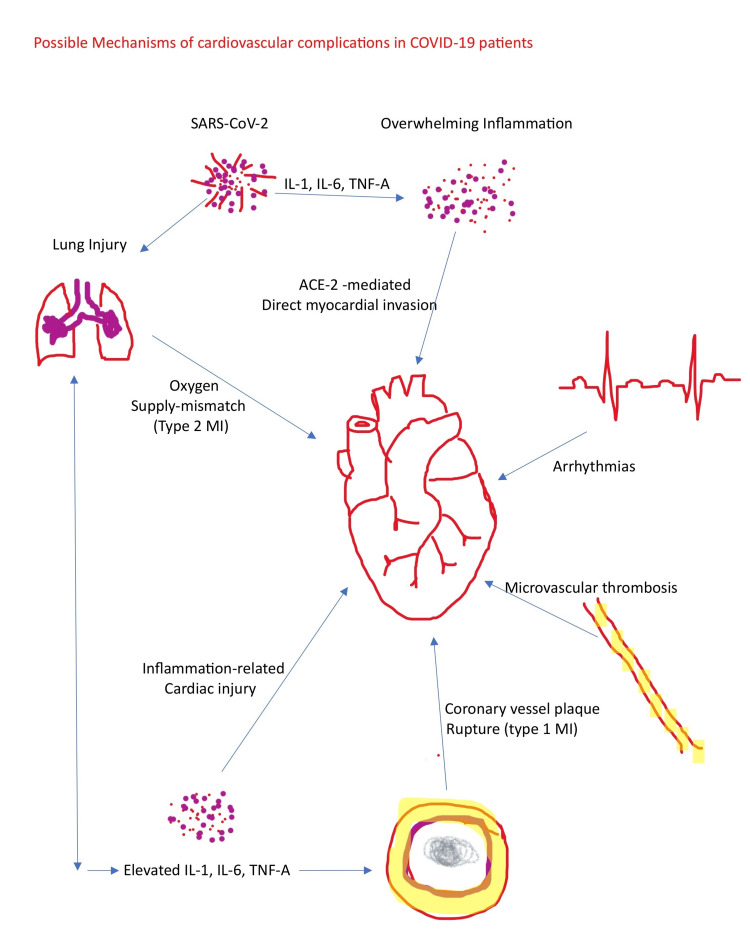
Possible path-physiology of COVID-19-induced cardiovascular complications

Several direct and indirect mechanisms have been used to explain the possible link between COVID-19 and cardiovascular complications (Figure 2). A complex interaction between the virus, the host responses, and underlying cardiovascular comorbidities characterize the link. Cardiac injury results from an interplay between the virus and host cells [96]. Studies from autopsy reports have also shown macrophage and lymphocytic infiltration, as well as viral fragments in the endothelium of cardiac vessels; however, no viral replication was noted [97]. Also, enormous inflammatory changes have been reported through cytokine storms and dysregulation of the host immune response [98]. Hypoxia from COVID-19-related respiratory failure has also led to an oxygen supply/ demand mismatch, further increasing the risk for myocardial infarction [99]. There is also a possible imbalance between the pro- and anti-inflammatory system resulting in an erratic inflammatory response activation, including catecholamine and IL-6 release, and the consequent inability of the host to limit inflammation [100, 101]. This further leads to overwhelming inflammation. The release of catecholamines increases heart rate and oxygen demand, which is detrimental to cardiac function. The overwhelming release of inflammatory markers (cytokines and chemokines) also leads to endothelial dysfunction, spasms of coronary arteries, and thrombi formation, which to a greater extent reduces blood supply to the heart. The above mechanisms: hypoxia and inflammation, leading to mitochondrial dysfunction, and alterations of calcium channels leading to impairment in myocytes' contractility activity [102]. Hypercoagulation through enormous inflammation has also been noted in COVID-19 patients, potentially responsible for some cardiac injuries, such as occlusive thrombus formation [103]. Drug-related cardiac injuries have also been reported, especially QT prolongation from medications (hydroxychloroquine, azithromycin) used to treat COVID-19 infection [104].

COVID-related acute cardiac injury

Type 1 Myocardial Infarction

One of the most evident/common/reported complications of COVID-19 infection is acute myocardial infarction, with several cases of COVID-19-related myocardial infarction reported in the literature, as noted in Table 1. Most of the patients reported were symptomatic with typical angina symptoms. Hypertension, diabetes, a history of coronary artery disease, and smoking were these patients' most commonly reported comorbidities [8, 11, 12-14, 17-20, 23-26, 29]. However, some cases were reported in patients without medical history [9, 10]. Although this complication was more common in patients older than 50 years, there were a few cases in the younger population, especially those with severe COVID-19 infection [9, 10]. At least two coronary dissection-induced myocardial infarction cases have also been reported; however, this was more common in patients with no documented medical history [34, 35]. Possible pathophysiology is secondary to the overwhelming release of inflammatory markers (cytokines and chemokines), which leads to endothelial dysfunction, coronary artery spasm, and thrombosis [99]. Also, the release of catecholamines increases heart rate and oxygen demand, which is detrimental to cardiac function. The increased risk of hypercoagulation in severe cases of COVID-19 is another mechanism that has been suggested [103]. There were also varying outcomes, with better outcomes in younger patients with no reported past medical history [9,10]. Diabetes, hypertension, and smoking were the common comorbidities in patients who died. Early treatment and intervention remain effective in preventing fatal outcomes in patients with COVID-19-induced acute myocardial infarction.

COVID-19-Induced Heart Failure, Cardiogenic Shock

Acute heart failure is another documented cardiovascular complication of COVID-19 infection, as noted in Table 2. This is likely secondary to hypoxia and inflammation, leading to mitochondrial dysfunction and alterations of calcium channels leading to impairment in myocytes' contractility [102]. About 23% of 191 inpatients from Wuhan, China, developed new-onset heart failure [92]. Acute heart failure was more common in patients with pre-existing cardiovascular disease and associated with increased mortality [16, 33, 36, 38, 39]. However, there was at least one case of heart failure in a patient without documented past medical history [48]. A recent retrospective study also noted an increased risk of acute heart failure in diabetic patients (25.2%) versus non-diabetics (5.6%) which could suggest a possible association [16]. A case of right ventricular dysfunction was also reported, with a potentially worse outcome [39].

COVID-19-Induced Myocarditis

Myocarditis is an inflammatory condition that involves the heart muscles (myocardium), leading to a myriad of clinical manifestations: chest pain, irregular heartbeats, and difficulty with breathing. Like other viruses, myocarditis is a commonly reported cardiac complication of COVID-19, with several cases reported in the literature, as reported in Table 3. Myocarditis was more frequently seen in patients with a severe presentation of COVID-19, with an increased mortality risk. The first case of myocarditis was in a 63-year-old male with no significant past cardiac medical history who was noted to have elevated interleukin-6 (IL-6) and myocardial injury markers such as troponin I, who subsequently died [43]. A retrospective study by Finn et al. reported a mortality rate as high as 61% in patients with acute myocarditis [46]. Although more common in patients with a documented medical history, there were several cases of myocarditis in patients without significant medical history, especially in the younger population [40, 42, 45, 47]. Patients without underlying medical problems were also associated with better outcomes [19, 40, 42, 45]. A study also showed a possible association between diabetes and the risk of myocarditis in COVID-19 patients, with 36.6% of diabetic patients developing acute myocarditis versus 15.5% of non-diabetics [16]. Increased mortality was also observed in patients with fulminant myocarditis, often complicated by pericarditis, pericardial effusion, and cardiac tamponade [41]. Ismayl et al. also reported a possible progression to cardiogenic Shock in fulminant cases [41]. The potential link with cardiogenic Shock involves myocardial inflammation leading to an abrupt decrease in cardiac contractility, inotropic deficit, and subsequent increase in filling pressures and diffuse myocardial edema. Patients who recovered were those that responded well to anti-inflammatory agents such as colchicine, steroids, and intravenous immunoglobin (IVIG). This finding could suggest an inflammatory pathway as a cause of this presentation [42]. The main mechanisms discussed were cardiomyocyte destruction due to viral entry, cytokine release syndrome, and hyperinflammation [42].

COVID-Induced Pericarditis, Pericardial Effusion, and Cardiac Tamponade

Alongside type 1 myocardial infarction, pericardial-related complications are among the most reported cardiovascular complications of COVID-19 infection, as noted in Table 4. As with other cardiovascular complications, pericardial-related complications were more common in older patients and patients with existing cardiovascular comorbidities. However, at least two cases were noted in the younger population, both presenting with severe complications - massive hemorrhagic pericardial effusion and myopericarditis, respectively and associated with mortality [54, 59]. The recovered patients responded well to anti-inflammatory agents, which suggests underlying inflammation as the cause of this complication.

COVID-19-Induced Thromboembolism

Hypercoagulation through enormous inflammation has also been noted in COVID-19 patients, as noted in Table 5, potentially responsible for some cardiac injuries, such as occlusive thrombus formation [103]. The possible mechanism involves both direct (micro vasculitis viral damage) and indirect mechanisms (through downregulation of ACE2 receptor, hypoxia, and disseminated intravascular coagulopathy) and even from prolonged immobilization in severe COVID-19 cases [103]. There are at least nine case reports on COVID-induced thrombosis; common to these patients was existing medical history of diabetes, which could suggest a positive association [23, 60, 61, 63, 64, 67-70]. A significantly elevated D-Dimer level is also common among these patients. Among the reported complications were deep venous thrombosis and acute pulmonary embolism, which appears to be the most common - at least two cases of critical limb ischemia leading to amputation [60, 67]. A study by Katsoularis et al. revealed the odds ratio of stroke in COVID-19 patients is 3.63 (1.69 - 7.80) [66]. In a prospective study by Mónica et al., 17 (5.6%) of COVID-19 patients developed acute pulmonary embolism [21]. This is similar to the findings by Corrado et al., where 7.7% of COVID-19 patients developed thromboembolism. A retrospective study also reported disseminated intravascular coagulopathy in which 71.4% of non-survivors developed the complication vs. 0.6% of survivors [68]. A potentially severe complication of COVID-19-induced thromboembolism is myocardial infarction, reported in at least three case reports [60, 69, 70]. There were also at least three cases of mortality from the nine cases reported [62, 67, 70]. Cases with critical limb ischemia also ended with amputation [61, 67]. This suggests the possible need for anticoagulation in patients with severe presentations of COVID-19 and significantly elevated D-Dimer levels due to the hypercoagulability risk.

COVID-19-Induced Takotsubo Syndrome

Takotsubo syndrome has been identified as one of the most common cardiac complications of COVID-19, as noted in Table 6. In the cases reported, the patients presented with chest discomfort, elevated troponins, and EKG alterations. In the case series by Arroyo-Rodriguez et al., four of the cases demonstrated classic apical ballooning, with one patient showing atypical presentation with anterolateral akinesia [72]. Coronary angiography was done in 4/5 cases to rule out acute coronary syndrome, which came back negative.

Takotsubo syndrome was also found in older patients over 65; however, there was a case of a 53-year-old patient with stage III chronic kidney disease (CKD) [75]. A common complication of Takotsubo syndrome identified was a cardiogenic shock, which was also associated with an increased risk of mortality [72, 75]. In the case series by Arroyo-Rodriguez et al., there was mortality in 4/5 cases, contradictory to other reports with a survival rate of 91.6% [72]. A possible association was the severity of the COVID-19 infection, with increased mortality noted in patients that are mechanically ventilated due to acute respiratory distress syndrome (ARDS), older patients, need for vasopressors, underlying renal failure, and underlying heart failure [72]. The suggested link between COVID-19 and Takotsubo syndrome is an overactive immune response due to cytokine storm, sympathetic drive, and microvascular dysfunction [72]. This is similar to the exact mechanism identified in other cardiac complications of COVID-19.

COVID-19-Induced Arrhythmias

Alongside type 1 myocardial infarction, arrhythmias are a commonly seen cardiac complication and one of the early clinical manifestations of COVID-19, reported in the literature as noted in Table 7. Possible suggested etiologies are hypoxia, inflammatory cytokine storm, and drug interactions. In multiple studies on the cardiac complications of COVID, dysrhythmias/arrhythmias were the most common complication identified [22, 79]. New-onset atrial fibrillation appears to be the most frequent arrhythmia reported. In a recent retrospective study by Khawaja et al., 19.1% of COVID-19 patients developed new-onset atrial fibrillation [81]. This is similar to another retrospective study that revealed new-onset atrial fibrillation in 12.7% of the COVID-19 patients studied. Also, in a study by Mónica et al., 6.5% of the patients developed new-onset atrial fibrillation [21].

Conduction abnormalities were another commonly reported arrhythmia [11, 23, 77, 84, 85, 88]. In a case by Lalani et al., 45.6% of 730 patients developed prolonged QTc interval, while 24.2% developed sinus tachycardia [11]. A possible explanation is using drugs such as azithromycin and hydroxychloroquine in COVID-19 patients [104]. Wide complex tachyarrhythmias were also reported, with cases of ventricular tachycardia - both sustained and non-sustained, ventricular fibrillation [77, 83]. Patients with underlying cardiovascular diseases were also noted to be at an increased risk of new-onset arrhythmias. In a study by Abe et al., 12.7% had new-onset atrial fibrillation versus 1.4% of non-diabetics [16].

Bradycardia is a crucial possible complication of COVID-19 infection that is frequently reported in the literature [23, 77, 82]. In a case series by Kaeley et al., 25% of patients developed a new-onset right bundle branch block [23]. The emergence of bradyarrhythmias in COVID-19 patients has also been suggested as a possible complication of an extreme cytokine storm with the potential for more serious cardiac complications [100, 101]. Guo et al., in a recent study, correlated an increase in N-Terminal proB-type Natriuretic Peptide (NP-proBNP) level with the development of malignant arrhythmias, which suggests a possible relationship between acute myocarditis and arrhythmias [95]. Therefore, it is essential to consider myocarditis in patients with a new development of tachyarrhythmias and increased cardiac biomarkers in patients with COVID-19.

## Conclusions

Acute cardiac injury is one of the most common complications of COVID-19 infection. Patients with acute cardiovascular complications appear to have pre-existing cardiovascular disease, older age group, and those with severe COVID-19 presentation. These groups also appear to have an increased risk of mortality. Type 1 myocardial infarction and arrhythmias appear to be the most common acute cardiac complication of COVID-19 infection, with hypertension and diabetes noted as one of the most common co-morbidities in these patients. This article provides an updated review of the acute cardiac complications of COVID-19. It also guides the suspicion of providers and serves as a future template for further research on each of the acute cardiac complications of COVID-19 infection, as well as providing support for implementing preventive strategies for patients at risk. Further studies are needed to evaluate the long-term cardiac complications of COVID-19.
